# Clinical Evaluation Based on a New Approach to Improve the Accuracy of 4β-Hydroxycholesterol Measurement as a Biomarker of CYP3A4 Activity

**DOI:** 10.3390/molecules28041576

**Published:** 2023-02-07

**Authors:** Yuki Taya, Mari Mizunaga, Shunsuke Nakao, Mirinthorn Jutanom, Naoki Shimizu, Yukihiro Nomura, Kiyotaka Nakagawa

**Affiliations:** 1 Laboratory of Food Function Analysis, Graduate School of Agricultural Science, Tohoku University, Sendai 980-8572, Miyagi, Japan; 2 Drug Metabolism and Pharmacokinetics Research Laboratories, Central Pharmaceutical Research Institute, Japan Tobacco Inc., Takatsuki 569-1125, Osaka, Japan

**Keywords:** biomarkers, cholesterol oxidation, cytochrome P450 CYP3A4 inducers, drug–drug interaction study, 4β-hydroxycholesterol, mass spectrometry

## Abstract

This study examines 4β-Hydroxycholesterol (4β-HC), which is considered to be a potential marker for the CYP3A4 induction of new chemical entities (NCEs) in drug development. To ensure the use of 4β-HC as a practical biomarker, it is necessary to accurately measure 4β-HC and demonstrate that CYP3A4 induction can be appropriately assessed, even for weak inducers. In clinical trials of NCEs, plasma is often collected with various anticoagulants, in some cases, the plasma is acidified, then stored for an extended period. In this study, we examined the effects of these manipulations on the measurement of 4β-HC, and based on the results, we optimized the plasma collection and storage protocols. We also found that a cholesterol oxidation product is formed when plasma is stored, and by monitoring the compound, we were able to identify when plasma was stored inappropriately. After evaluating the above, clinical drug–drug interaction (DDI) studies were conducted using two NCEs (novel retinoid-related orphan receptor γ antagonists). The weak CYP3A4 induction by the NCEs (which were determined based on a slight decline in the systemic exposure of a probe substrate (midazolam)), was detected by the significant increase in 4β-HC levels (more specifically, 4β-HC/total cholesterol ratios). Our new approach, based on monitoring a cholesterol oxidation product to identify plasma that is stored inappropriately, allowed for the accurate measurement of 4β-HC, and thus, it enabled the evaluation of weak CYP3A4 inducers in clinical studies without using a probe substrate.

## 1. Introduction

**Figure 1:**
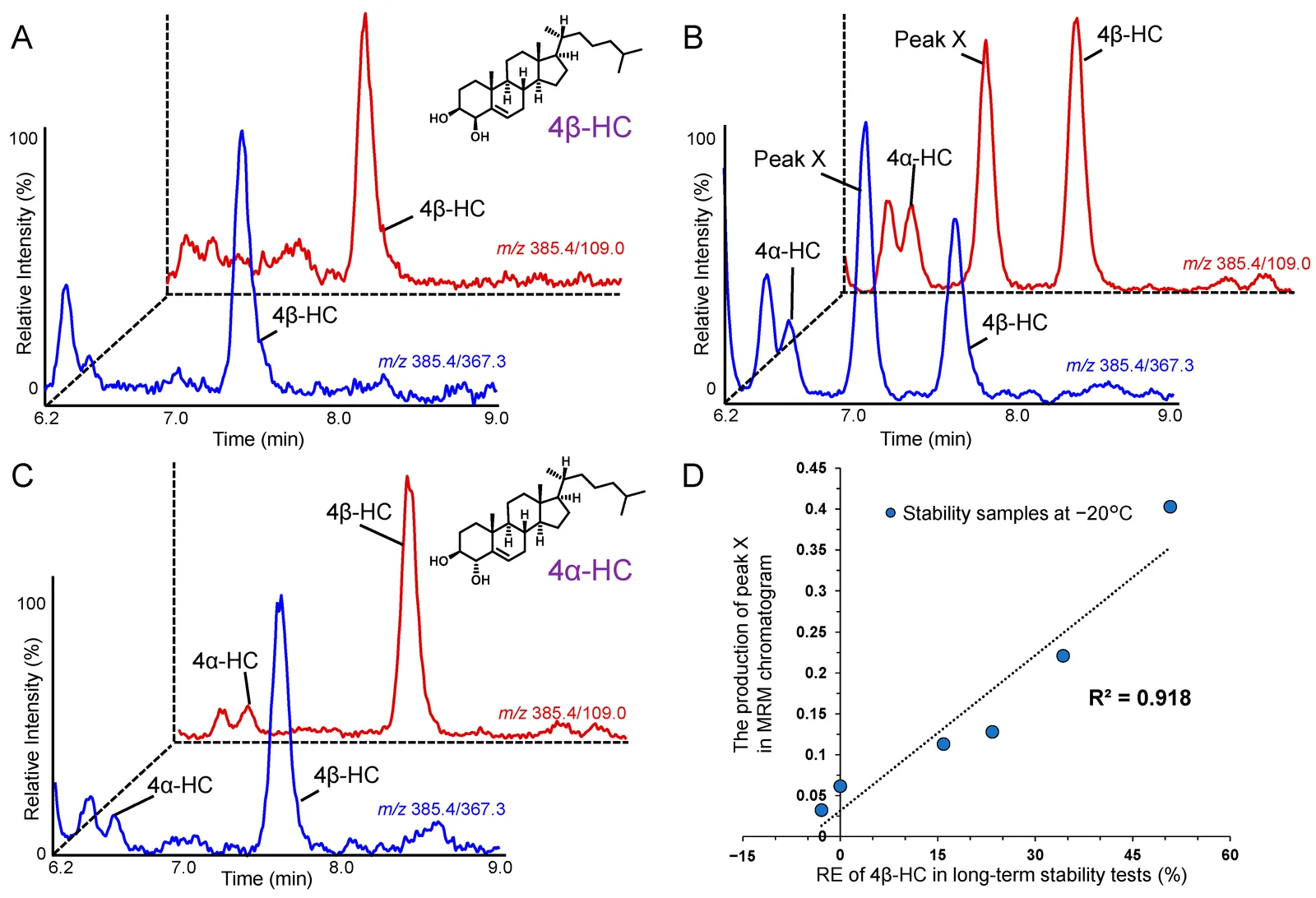
Typical MRM chromatograms analyzing plasma that was obtained with K2-EDTA, then analyzed without storage (**A**), or analyzed after 13 months of storage in the dark at −20 °C (**B**) or −80 °C (**C**). Each plasma sample was saponified, extracted with hexane, and analyzed with LC-MS/MS in the MRM mode. Extended MRM chromatograms between the retention times of 6.2–9.0 min are shown. Other details are described in the Methods section. Identification of 4β-HC and 4α-HC was performed by comparing the two markers against commercial standards. The correlation chart between the production of peak X and the relative error (RE) of 4β-HC in the plasma was analyzed during the long-term storage test (**D**). Peak X was corrected in accordance with the internal standard 4β-HC-d7 (i.e., the ratio of the area of peak X to the area of 4β-HC-d7 was calculated). A correlation diagram was created by plotting the peak area ratio of peak X and 4β-HC-d7 after long-term storage at −20 °C on the Y-axis and the RE shown in Table 1B on the X-axis (**D**).

Cytochrome P450 family 3 subfamily A member 4 (CYP3A4) plays a role in the metabolism (oxidation) of cholesterol to 4β-hydroxycholesterol (4β-HC, Figure 1A) [1,2]. In support of this, many clinical studies have reported that the administration of CYP3A4 inducers (e.g., rifampicin, a strong inducer of CYP3A4) increases the concentration of 4β-HC (or 4β-HC/total cholesterol ratio (4β-HC/TC ratio)) in human plasma [3,4,5,6,7]; thus, 4β-HC is considered a potential marker for assessing the risk concerning whether a new chemical entity (NCE) induces CYP3A4 [8]. Although several clinical studies have assessed the above possibility using strong inducers of CYP3A4 (e.g., rifampicin) [3,4,5,6,7], further clinical studies, with various CYP3A4 inducers (especially weak inducers), are required.

To correctly evaluate the relationships between CYP3A4 inducers and plasma 4β-HC levels, an accurate analysis of 4β-HC in plasma is necessary. Factors that may hinder accurate analysis include plasma collection conditions and storage conditions [9]. From this perspective, the acidification of plasma (obtained using various anticoagulants) is known to provide a stable measurement of NCEs [10,11]. Nevertheless, the effects of anticoagulants (except K2-EDTA) and acidic conditions on the analysis of 4β-HC in plasma have not been properly evaluated [12,13,14,15]. In addition, as various factors (e.g., storage conditions and collection conditions) influence the quality of plasma samples [9], knowledge pertaining to indicators of defective plasma samples (i.e., inappropriately stored samples) is also desirable. In this regard, previous studies have proposed that plasma sample quality is likely to be compromised if the plasma concentration of 4α-hydroxycholesterol (4α-HC, Figure 1C), an isomer of 4β-HC, is remarkably higher than that of basal concentrations [16,17]. Other indicators that are more sensitive when detecting defective plasma should enhance the accuracy of 4β-HC quantitation in plasma and enable a more detailed evaluation of the relationships between CYP3A4 inducers and plasma 4β-HC levels.

To this end, we first assessed the stability of 4β-HC in plasma/serum collected with common anticoagulants (e.g., sodium heparin) and acidic solutions. Next, we confirmed that a cholesterol oxidation compound other than 4α-HC was produced during the storage of plasma samples, thus suggesting that the monitoring of the compound allows the detection of plasma samples stored in improper conditions. Based on the above, appropriate plasma collection conditions, and an indicator that can detect defective plasma samples, were investigated. We further evaluated the effect that weak inducers of CYP3A4 have on 4β-HC levels in plasma. More specifically, we conducted clinical drug–drug interaction (DDI) studies using two in-house developed NCEs (JTE-451 and JTE-761 [18], which were both weak inducers of CYP3A4) and midazolam, a probe substrate for CYP3A4 activity [19]. Repeated administration of JTE-451 and JTE-761 slightly decreased the area under the curve (AUC) for midazolam, thus confirming that both JTE-451 and JTE-761 were, indeed, weak CYP3A4 inducers. Additionally, we found that the plasma 4β-HC/TC ratio, rather than the plasma concentration of 4β-HC, demonstrates a strong negative correlation with the AUC of midazolam. These results suggest that 4β-HC/TC ratios could be used as a marker to determine the strength of CYP3A4 induction (even when induced by weak inducers) by rigorously controlling the conditions of the plasma samples.

**Table 1:** Long-term storage stability test of human plasma, serum, and acidified plasma.

A								B			C	
Storage Conditions Temperature/Period	Human Plasma				Acidified Human Plasma		Human Serum	Storage Conditions Temperature/Period	Human Plasma		Storage Conditions Temperature/Period	Human Plasma
	K2-EDTA	Heparin Sodium	Heparin Lithium	3.2% Sodium Citrate	K2-EDTA Containing Acetic Acid	Heparin Sodium Containing Acetic Acid			K2-EDTA	Heparin Sodium		K2-EDTA
Before storage	27.9	28.3	27.0	23.6	26.6	27.5	28.4	Before storage	32.5	30.0	Before storage	35.2
−20 °C/1 month	25.3 ± 0.6	26.5 ± 0.6	28.5 ± 1.8	22.6 ± 1.2	26.8 ± 1.3	25.9 ± 1.6	27.8 ± 2.2	−20 °C/6 months	30.3 ± 2.2	30.0 ± 1.6	−70 °C/1 month	35.3 ± 0.5
	(−9.3)	(−6.4)	(5.6)	(−4.2)	(0.8)	(−5.8)	(−2.1)		(−6.8)	(0.0)		(0.3)
−20 °C/2 months	26.2 ± 2.4	25.2 ± 2.7	26.4 ± 0.8	22.2 ± 1.2	25.4 ± 2.0	25.9 ± 1.6	26.3 ± 0.9	−20 °C/9 months	37.4 ± 0.2	35.8 ± 0.9	−70 °C/3 month	33.4 ± 0.5
	(−6.1)	(−11.0)	(−2.2)	(−5.9)	(−4.5)	(−5.8)	(−7.4)		(* 15.1)	(* 19.3)		(−5.1)
−20 °C/6 months	30.9 ± 1.2	29.2 ± 2.4	30.7 ± 1.4	24.9 ± 0.9	31.0 ± 0.7	41.2 ± 1.2	28.2 ± 3.7	−20 °C/13 months	45.7 ± 0.9	41.5 ± 0.5	−70 °C/10 month	35.7 ± 1.2
	(10.8)	(3.2)	(13.7)	(5.5)	(* 16.5)	(* 49.8)	(5.6)		(* 40.6)	(* 38.3)		(1.4)
−80 °C/1 month	25.5 ± 1.0	26.1 ± 0.8	28.0 ± 0.2	24.2 ± 0.9	26.5 ± 1.0	26.5 ± 0.5	29.7 ± 1.0	−80 °C/6 months	29.5 ± 1.2	29.1 ± 1.1		
	(−8.6)	(−7.8)	(3.7)	(2.5)	(−0.4)	(−3.6)	(4.6)		(−9.2)	(−3.0)		
−80 °C/2 months	25.7 ± 1.4	26.0 ± 0.6	24.7 ± 0.8	22.3 ± 0.7	27.2 ± 0.5	26.2 ± 1.3	25.7 ± 1.7	−80 °C/9 months	32.0 ± 1.0	30.9 ± 1.3		
	(−7.9)	(−8.1)	(−8.5)	(−5.5)	(2.3)	(−4.7)	(−9.5)		(−1.5)	(3.0)		
−80 °C/6 months	29.7 ± 0.6	28.7 ± 1.2	28.3 ± 0.7	24.2 ± 0.8	28.7 ± 0.6	29.4 ± 0.3	29.0 ± 0.4	−80 °C/13 months	33.8 ± 1.4	33.1 ± 1.3		
	(6.5)	(1.4)	(4.8)	(2.5)	(7.9)	(6.9)	(2.1)		(4.0)	(10.3)		

Plasma or serum was prepared using blood that was collected from six healthy volunteers (three males and three females who did not take the test drug) using various anticoagulants (K2-EDTA, heparin sodium, heparin lithium, or 3.2% sodium citrate) or a serum-separating medium. Each sample was saponified and extracted with hexane immediately after collection. In the extracts, 4β-HC was quantified with LC-MS/MS, as described in the Methods section. The mean 4β-HC concentrations of six volunteers before storage are shown. The 4β-HC concentrations of each sample were similar to those reported in previous studies [2,20]. The plasma (or serum) of the six volunteers were then pooled into one, and the combined plasma was then subjected to the following storage test: storage at −20 °C or −80 °C in the dark for up to 6 months (**A**) or up to 13 months (**B**). K2-EDTA plasma was stored at −70 °C in the dark for up to 10 months, thus mimicking the storage conditions of clinical testing sites (**C**). The 4β-HC concentrations in the samples that were subjected to the storage test were measured in triplicate, and the results are expressed as mean ± standard deviation (SD). The relative error (RE, in parentheses) was calculated via the difference in concentration of 4β-HC before and after storage, and RE was indicated with an asterisk (*) if it exceeded the acceptance criteria of ±15%.

## 2. Results and Discussion

### 2.1. Stability of 4β-HC in Plasma/Serum Collected with Various Anticoagulants and Acid Solutions

The ICH M10 guidelines [21] require that plasma NCEs and their metabolites are measured in clinical trials using appropriate protocols. To accurately measure NCEs and metabolites in accordance with these guidelines, plasma collected with various anticoagulants is often stabilized under acidic conditions to prevent artificial (ex vivo) conversions (e.g., metabolites of NCEs, such as acyl glucuronides, lose their glucuronic acid groups when plasma is stored under neutral conditions) during storage [10,11]; however, little is known about the effects of these anticoagulants, or plasma acidification, on the measurement of 4β-HC. Previous reports are limited to examining the stability of 4β-HC in plasma collected with K2-EDTA or in serum [12,13,14,15]; thus, we considered it necessary to evaluate the effects of various anticoagulants and plasma acidification on the measurement of 4β-HC under different plasma storage conditions.

Plasma collected during clinical trials is often frozen for long periods of time (several years in the longest case) before being analyzed [22]. Hence, we first examined the stability of 4β-HC during the long-term storage of plasma that was collected with various anticoagulants (i.e., K2-EDTA, heparin sodium, heparin lithium, and 3.2% sodium citrate). When plasma was stored at −20 °C and −80 °C, 4β-HC was stable for up to 6 months after collection with all the anticoagulants evaluated in this study (Table 1A). Moreover, when plasma obtained with K2-EDTA or heparin sodium was stored at −20 °C for more than 9 months, the concentration of 4β-HC significantly increased, presumably due to autoxidation (Table 1B) [14]; therefore, the antioxidative effects of anticoagulants (e.g., chelation of K2-EDTA) may be insufficient to store plasma for prolonged periods. Additionally, the results suggest that the type of anticoagulant has little influence on the accurate measurement of 4β-HC, and the storage of plasma (or serum) at −80 °C is acceptable; however, due to the occasional occurrence of freezer limitations at the clinical testing sites, the plasma samples were stored at −70 °C before being measured [23]. Thus, we further confirmed that 4β-HC was stable enough for long-term storage (up to 10 months) at temperatures under −70 °C with K2-EDTA (Table 1C); this means that the storage of plasma (or serum) at −70 °C is also acceptable. For reference, 4β-HC was stable in plasma stored at 4 °C, or at room temperature, with or without light for 6 h (data not shown), thus suggesting that 4β-HC is stable during sample preparation procedures. Moreover, 4β-HC was also stable for up to five freeze-thaw cycles at −20 °C and −80 °C (data not shown). We anticipate these data to be important for the accurate measurement of plasma 4β-HC concentrations.

Next, the effect of plasma acidification on the concentration of 4β-HC was examined. Plasma was collected using K2-EDTA or heparin sodium as an anticoagulant. Even under acidic conditions, no significant change in the concentration of 4β-HC was observed in plasma stored at −80 °C for up to 6 months (Table 1A); however, acidified plasma was more unstable compared with unacidified plasma that was stored at lower temperatures. For example, 4β-HC clearly increased when acidified heparin sodium plasma was stored at −20 °C for 6 months (Table 1A). Analyzing the mechanisms behind this increase in 4β-HC should a priority for future studies because, to the best of our knowledge, no study has reported the autooxidation of cholesterol under acidic conditions. Overall, the long-term storage of acidified plasma suggests that it is not desirable to store plasma under acidic conditions when aiming to accurately measure 4β-HC (i.e., plasma stored under acidic conditions to measure NCEs should not be used for the measurement of 4β-HC).

Based on the above results, plasma samples that were taken in order to measure 4β-HC in later clinical DDI studies were collected with K2-EDTA, without acidification, and separately from those that were taken to measure NCEs. The collected plasma samples were stored directly in a deep freezer set at −70 °C (clinical testing sites) or −80 °C (analytical laboratories). For shipment, samples were kept frozen at −79 °C using dry ice.

### 2.2. Indicators to Detect Defective Plasma That Contribute to Improving the Accuracy of 4β-HC Measurements in Plasma

As described above, we identified the protocols of plasma preparation and storage to accurately measure 4β-HC in plasma; however, even under these conditions, the deterioration of plasma due to unpredictable factors (e.g., opening and closing of freezers) is unavoidable [9]. Thus, an indicator that can detect such defective plasma is useful to ensure the quality of the plasma. As mentioned in the introduction, previous studies have noted that the plasma concentration of 4α-HC (an isomer of 4β-HC) increases when plasma is stored, and thus, 4α-HC may be a suitable indicator that can detect defective plasma [16,17]. To confirm this, plasma obtained with K2-EDTA was stored at −20 °C for 13 months and analyzed using LC-MS/MS. As shown in Figure 1, 4α-HC was indeed detected, along with 4β-HC; however, the increase in 4α-HC when the plasma was stored at −20 °C was not significant, and the correlation between the relative error (RE) and the increase in 4β-HC during storage was weak (data not shown). Therefore, we considered it necessary to find another indicator that was more sensitive than 4α-HC for assessing plasma quality.

**Figure 2:**
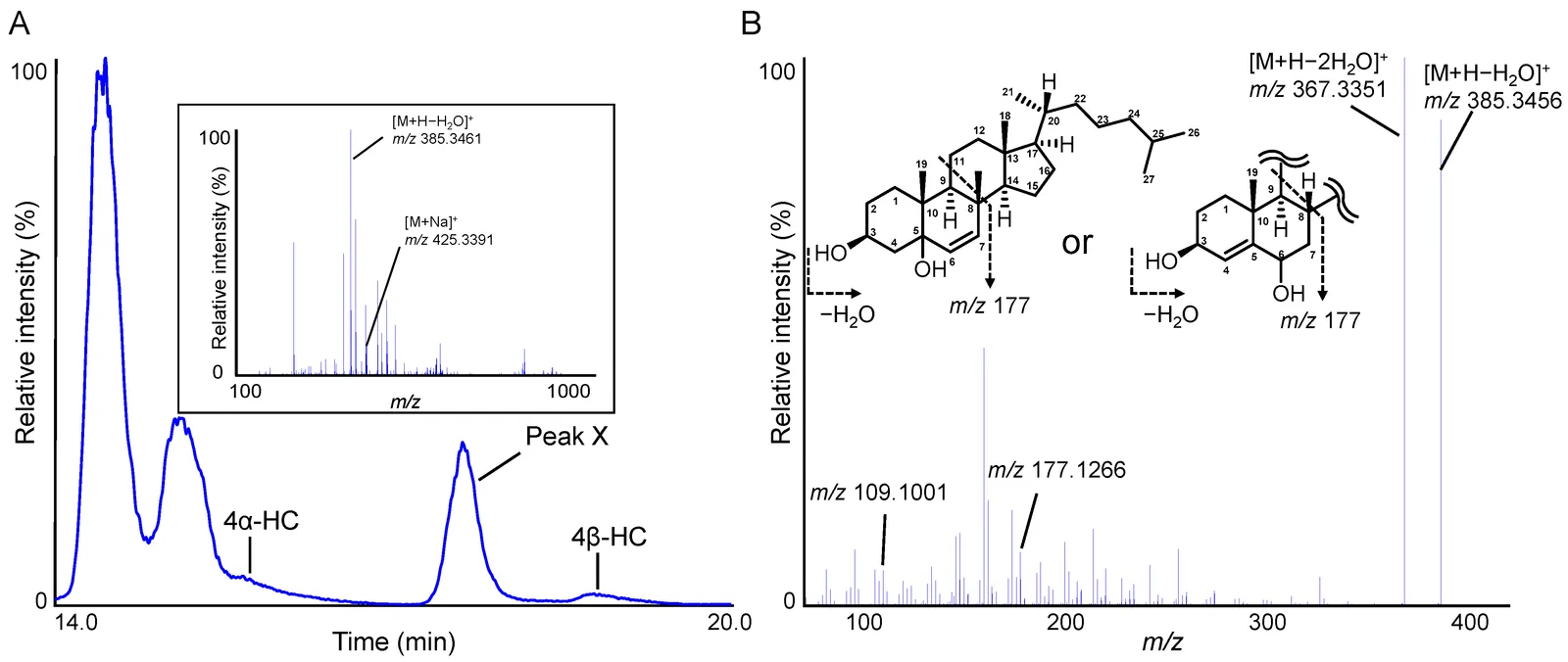
Typical Q1 chromatogram and MS spectrum of peak X (**A**); product ion spectrum of peak X (**B**). An extended Q1 chromatogram between the retention times of 14.0–20.0 min is shown. K2-EDTA plasma collected from healthy volunteers, stored at −20 °C for 13 months in the dark, was analyzed by LC-HRMS. Details of this process are shown in the Methods section. The sample was saponified, extracted with hexane, and analyzed using LC-HRMS. Refer to the Methods section for details regarding the conditions.

Under these circumstances, we noticed that peak X, in the chromatogram of stored plasma (Figure 1D), correlated well with the RE relating to the increase in 4β-HC during storage (correlation coefficient of 0.918). Such a high correlation suggested that peak X is an appropriate indicator of defective plasma. As peak X was detected under the same MRM pairs that were used to detect 4β-HC and 4α-HC (e.g., *m*/*z* 385.4/109.0 and *m*/*z* 385.4/367.3), we presumed that peak X was also a cholesterol oxidation product (Figure 1B). To further analyze its structure, peak X was analyzed with LC-HRMS. Peak X (RT 17.6 min) formulates precursor ions at *m*/*z* 385.3461 [M+H−H_2_O]^+^ and *m*/*z* 425.3391 [M+Na]^+^, which corresponded with C_27_H_45_O (∆−1.0 ppm) and C_27_H_46_O_2_Na (∆−1.2 ppm), respectively (Figure 2A). These spectra suggested that peak X was an isomer of cholesterol hydroxide. MS/MS analysis of *m*/*z* 385.4 showed a distinct product ion at *m*/*z* 177.1266, which corresponded with C_12_H_17_O (Δ−4.5 ppm) (Figure 2B). This implied that the hydroxyl group was bound to the A or B ring of the steroid backbone. As the retention time of peak X did not match that of commercially available cholesterol hydroxide standards (4β-HC, 4α-HC, 7α-HC, and 7β-HC), nor that of related compounds (5,6α-epoxy cholesterol and 5,6β-epoxy cholesterol), presumably, the hydroxyl group of peak X is attached to the C5 or C6 position (data not shown). Indeed, previous studies have shown that the free radical-mediated oxidation (autoxidation) of cholesterol produces 4α-HC, 4β-HC, 5α-HC, 5β-HC, 6α-HC, 6β-HC, 7α-HC, and 7β-HC [24,25,26]. Their expected formation pathways have been reported in previous studies [25,26]. Further studies are needed to determine the exact structure of peak X (i.e., whether the hydroxyl group is bound to the C5 or C6 position). Based on these results, in the following DDI studies, we decided to exclude plasma samples in which peak X was detected during analysis.

### 2.3. DDI Studies to Evaluate Whether Weak Inducers of CYP3A4 (JTE-451 and JTE-761) Affect Plasma 4β-HC Levels and the AUC of Midazolam

**Figure 3:**
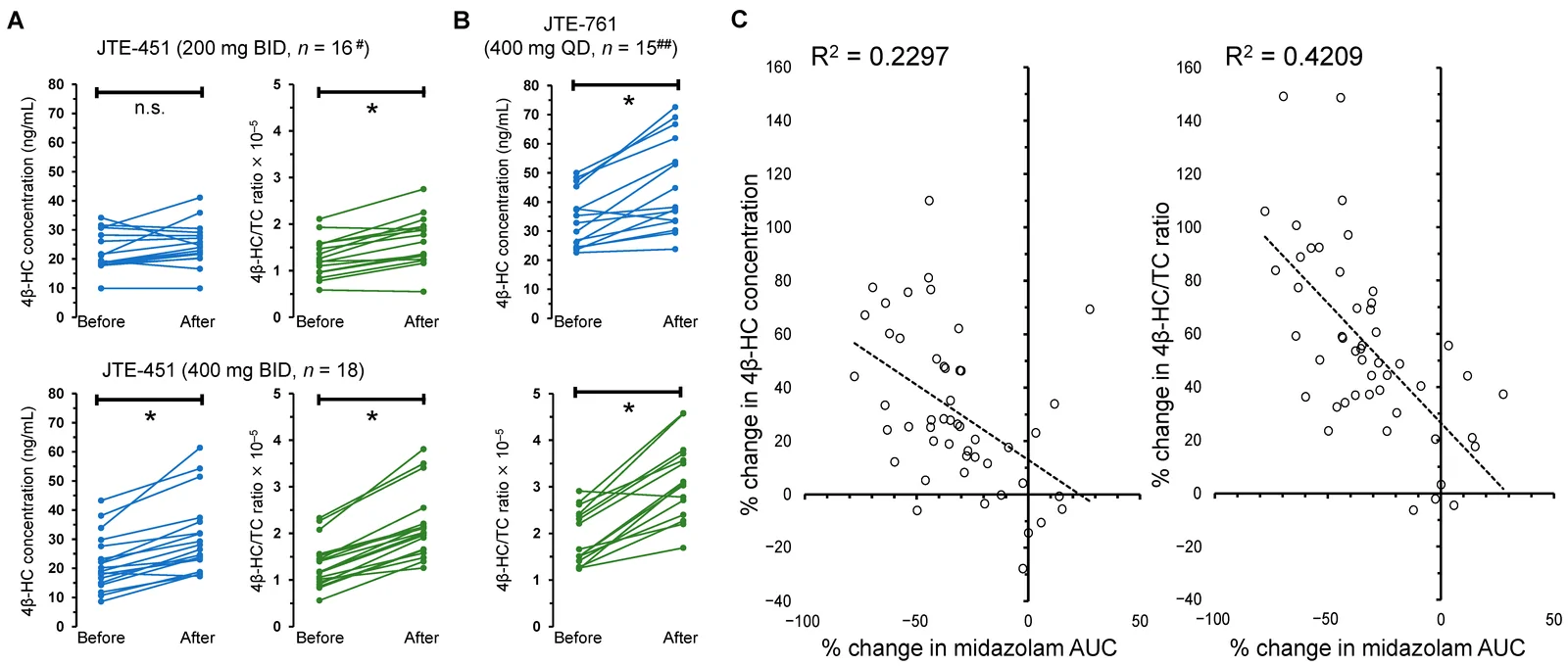
The 4β-HC concentrations and 4β-HC/TC ratios before and after the repeated administration of JTE-451 (**A**) and JTE-761 (**B**). The correlation chart between plasma 4β-HC concentrations or 4β-HC/TC ratios and the AUC of midazolam (**C**). Clinical DDI studies were conducted using weak inducers of CYP3A4 (JTE-451 and JTE-761), and blood collection and plasma sample storage were performed based on the results of 4β-HC stability tests. Midazolam and 4β-HC were measured using plasma, and the total cholesterol was measured using serum. The percentage (%) changes in 4β-HC concentration, 4β-HC/TC ratio, and midazolam AUC demonstrate their differences before and after the administration of NCEs. ^#^ Two subjects out of eighteen subjects opted out of the clinical study. ^##^ One subject out of sixteen subjects opted out of the clinical study. * *p* < 0.01, n.s.: not significant.

Two DDI studies were conducted under the conditions evaluated above. Using two in-house developed NCEs (JTE-451 and JTE-761 [18], which are weak inducers of CYP3A4) and midazolam (a probe substrate for CYP3A activity [19]), we first evaluated whether the administration of these weak inducers increases plasma 4β-HC concentrations. A significant increase in plasma 4β-HC concentrations were observed only when JTE-451 and JTE-761 were administered at 400 mg BID and 400 mg QD, respectively (Table 2 and Figure 3). Interestingly, 4β-HC/TC ratios were significantly elevated at all dosage levels of JTE-451 and JTE-761 (Table 2 and Figure 3). Similarly, previous studies have reported that 4β-HC/TC ratios fluctuate when CYP3A4 inducers are administered, although no study has been conducted for weak CYP3A4 inducers, to the best of our knowledge [4,5,6,7,27]. As cholesterol concentrations influence the generation of 4β-HC [16,28], we believe that 4β-HC/TC ratios are more suitable than 4β-HC concentrations when plasma cholesterol concentrations fluctuate, as was the case in this study (Table 2); however, further clinical studies are needed to demonstrate that the use of 4β-HC/TC ratios are more practical for assessing CYP3A4 induction than 4β-HC concentrations, as there were certain limitations to this study (e.g., no placebo groups were set).

For the assessment of CYP3A4 induction, NCEs were ranked in accordance with the criteria described in the FDA guidance on Clinical Drug Interaction Studies (2020) [29]. NCEs were ranked as strong inducers when the AUC of midazolam decreased by ≥80% upon administration, moderate when the AUC decreased by ≥50% to <80%, and weak when the AUC decreased by ≥20% to <50%. As a result, 400 mg BID of JTE-451 and 400 mg QD of JTE-761 were classified as weak inducers, whereas 200 mg BID of JTE-451 was classified as a very weak inducer (Table 2). As JTE-451 and JTE-761 were confirmed to be weak inducers, we compared the AUC of midazolam with the plasma concentration of 4β-HC (or 4β-HC/TC ratio) after the administration of each NCE. As a result, the increase in 4β-HC/TC ratios, but not the increase in 4β-HC concentrations, significantly correlated with the decrease of AUC in midazolam (Figure 3C). To our knowledge, although previous studies reported a 1.3-fold increase in the 4β-HC/TC ratio after the repeated administration of rifampicin at doses which had a medium inducing effect (10 mg) [4,5], no previous study has demonstrated an increase in plasma 4β-HC concentration or 4β-HC/TC ratio after the administration of a weak inducer in clinical DDI studies. Although the CYP3A4 induction ability of the NCEs used in the present study appears to be weaker than that of 10 mg rifampicin [5], their administration resulted in an increase for the 4β-HC/TC ratios by more than 1.3-fold. This may be attributed to the high precision with which 4β-HC was quantified in this study. These findings suggest that controlling the plasma’s sample conditions, and monitoring indicators of defective plasma samples, can provide an accurate assessment of CYP3A4 induction via the measurement of 4β-HC/TC ratios. The results also suggest that plasma 4β-HC/TC ratios may be a more suitable biomarker to assess CYP3A4 induction than 4β-HC concentrations, even for the assessment of weak inducers. It is also of note that plasma 4β-HC concentrations and 4β-HC/TC ratios were increased after the repeated administration of two NCEs, developed in-house, that possess a mechanism of action that is different from rifampicin [30]. Such results enhance the reliability of 4β-HC as a biomarker that can evaluate CYP3A4 induction potential.

## 3. Materials and Methods

### 3.1. Chemicals and Reagents

The 4β-HC, 4β-hydroxycholesterol-d7 (4β-HC-d7, an internal standard), and other oxysterols were purchased from Avanti Polar Lipids (Alabaster, AL, USA). The purity of the standards was >99%. The 4α-HC was obtained from Toronto Research Chemicals (Toronto, ON, Canada). Butylhydroxytoluene (BHT) was obtained from Sigma-Aldrich (St. Louis, MO, USA), and sodium methoxide was obtained from Tokyo Chemical Industry (Tokyo, Japan). All other chemicals were above the analytical grade.

### 3.2. Investigation of Plasma Collection Methods, Storage Conditions, and Indicators of Defective Plasma Samples

Blood samples were collected from healthy volunteers (three males and three females) into vacuum collection tubes (Terumo Corporation, Tokyo, Japan) containing either K2-EDTA (P/N VP-DK050K), heparin sodium (P/N VP-H100K), heparin lithium (P/N VP-HL050K), 3.2% sodium citrate (P/N VP-CA050K70), or a serum separating medium (P/N VP-AS109K). The tubes were centrifuged at 4 °C for 10 min at 1710× *g* to separate the plasma (or serum). Equal amounts of plasma (or serum) from each volunteer were pooled and dispensed into polypropylene microtubes. Acidified plasma, prepared by adding 1/10 volume of water/acetic acid (19:1, *v*/*v*) to K2-EDTA plasma or sodium heparin plasma, was also dispensed into tubes.

The sample tubes, each containing 600 μL of plasma or serum, were stored for long or short periods of time under various conditions to mimic actual 4β-HC storage conditions (e.g., the sample storage temperature in clinical testing sites and analytical laboratories, shipping conditions, and conditions during sample preparation procedures). For the long-term stability tests, tubes were stored in the dark at −20 °C or −80 °C for up to 13 months (the duration of stability tests: 1, 2, 6, 9, and 13 months) and at −70 °C for up to 10 months (the duration of stability tests: 1, 3, and 10 months). For the short-term stability tests, tubes were stored for 6 h under three conditions: in the dark (in a refrigerator, 4 °C), under fluorescent light (room temperature), and shading with aluminum foil (room temperature). For the freeze-thaw stability tests, tubes were frozen at −20 °C or −80 °C, thawed at room temperature for about 30 min, and then frozen for up to five cycles. After each test, 4β-HC was extracted from the plasma (or serum) and quantified via liquid chromatography-tandem mass spectrometry (LC-MS/MS), as described below. During LC-MS/MS analysis, we found a compound that was a potential indicator for defective plasma samples. Its structure was analyzed using LC-high-resolution mass spectrometry (LC-HRMS), as described below. Based on the results of these experiments, the optimal conditions for sample collection, storage, and shipping, when mimicking the conditions of 4β-HC storage at clinical testing sites and analytical laboratories, were determined.

### 3.3. Clinical DDI Studies of NCEs (JTE-451 and JTE-761)

Phase 1, open-label DDI studies of two novel retinoid-related orphan receptor γ antagonists, JTE-451 (AE451-U-19-005) and JTE-761 (AE761-U-19-001) [18], were conducted in 36 and 16 healthy adult male subjects, respectively. Thirty-six subjects were enrolled in two cohorts, wherein subjects received either a 200 or 400 mg twice-daily (BID) dose of JTE-451 (18 subjects per cohort). Sixteen subjects were enrolled in another cohort, wherein subjects received a 400 mg once-daily (QD) dose of JTE-761. All subjects received a single oral dose of 3 mg midazolam either 9 days or 1 day prior to the administration of JTE-451 or JTE-761, respectively. Then, JTE-451 or JTE-761 was administered for 14 consecutive days (days 1 to 14). On day 15, JTE-451 or JTE-761 was co-administered with midazolam. An overview of the dosing schedules of the test compounds are shown in Supplementary Figure S1.

Blood was collected before the administration of JTE-451 or JTE-761, and on the last day of the administration of JTE-451 or JTE-761. Plasma was collected and stored using the optimal method described in Section 2.2. Clinical studies, and experiments with the human samples, as described in Section 2.2, were conducted in accordance with the principles of the Declaration of Helsinki. The protocols were approved by the Institutional Review Board of IntegReview IRB, Austin, Texas. The studies were also conducted in accordance with the principles of Good Clinical Practice. All subjects provided written informed consent prior to participation in the study.

### 3.4. LC-MS/MS Analysis of 4β-HC in Plasma and Serum

A 100 μL aliquot of human plasma (or serum) was taken from the stability tests or clinical studies and mixed with 10 μL of 4β-HC-d7 (internal standard, 1 μg/mL in 2-propanol), 50 μL of BHT (antioxidant, 1 mg/mL in ethanol), 350 μL of Tween 80 (1% in phosphate-buffered saline (PBS)), and 1 mL of sodium methoxide (2 mol/L in methanol/ethanol (2:3, *v*/*v*)) [20,31]. Each sample was saponified at room temperature for 20 min, mixed with 4 mL of hexane and 500 μL of saturated NaCl solution, and held at room temperature for 15 min. After centrifugation (1710× *g* for 5 min at 4 °C), the organic layer was collected, dried, and dissolved in 100 μL of formic acid/acetonitrile/water (1:900:100, *v*/*v*/*v*). Samples were stored at 4 °C in the autosampler, and a 5 μL aliquot was injected into the LC-MS/MS system.

The LC-MS/MS system consisted of a Nexera X2 HPLC (Shimadzu, Kyoto, Japan) coupled with a Triple Quad 6500 mass spectrometer (AB Sciex, Framingham, MA, USA). An L-column2 ODS (150 mm × 2.1 mm ID, 2 μm, Chemical Evaluation and Research Institute, Tokyo, Japan) was used at 50 °C. A binary gradient consisting of mobile phase A (water/formic acid (1000:1), *v*/*v*) and B (acetonitrile/formic acid (1000:1), *v*/*v*) was employed under the following profile: 0–9.0 min, B 90%; 9.0–9.1 min, B 90–95%; 9.1–12 min, B 95%; 12.0–12.1 min, B 95–90%; 12.1–14.0 min, B 90%. The flow rate was set at 0.5 mL/min. MS/MS detection of the analytes was performed in the multiple reaction monitoring (MRM) mode with positive electrospray ionization. MRM pairs were *m*/*z* 385.4/109.0 for 4β-HC and *m*/*z* 392.4/109.0 for 4β-HC-d7. The de-clustering potential, collision energy, and collision exit potential were set at 136, 31, and 11 V, respectively. Other MS/MS parameters are described in Supplementary Table S1.

Calibration samples were prepared by spiking 10 μL of 4β-HC (0.05–2.5 μg/mL in 2-propanol) and 10 μL of 4β-HC-d7 (internal standard, 1 μg/mL in 2-propanol) into a surrogate matrix (100 μL of Tween 80 (1% in PBS)). Each sample was extracted using the same procedure as plasma (or serum) and analyzed using LC-MS/MS. A calibration curve for the quantification of 4β-HC in the plasma (or serum) samples was constructed by plotting the peak area ratio of 4β-HC to 4β-HC-d7 against their nominal concentrations (5–250 ng/mL).

### 3.5. LC-HRMS Analysis of a Potential Indicator to Detect Defective Plasma

The structure of the potential indicator to detect defective plasma, found during the long-term stability test, was analyzed using LC-HRMS. Plasma stored under the long-term storage test conditions (Section 3.4) was extracted, diluted, and subjected to LC-HRMS analysis. The LC-HRMS system consisted of a Nexera X2 HPLC, coupled with a TripleTOF 5600 (AB Sciex). Separation conditions (column, mobile phase, flow rate, and column temperature) for LC-HRMS analysis were the same as for the LC-MS/MS analysis described above. The LC gradient profile was as follows: 0–19.0 min, B 80%; 19.0–19.1 min, B 80–95%; 19.1–22.0 min, B 95%; 22.0–22.1 min, B 95–80%; 22.1–24.0 min, B 80%. HRMS parameters are shown in Supplementary Table S1.

### 3.6. Other Measurements in Clinical DDI Studies

For the measurement of TC levels, serum samples were collected before the administration of JTE-451 or JTE-761, and on the last day of the administration of JTE-451 or JTE-761, TC levels were measured using a colorimetric analysis kit.

Plasma samples collected from the subjects on midazolam administration dates, as described in Section 2.3, were used to analyze plasma midazolam concentrations. Midazolam was quantified using LC-MS/MS. Contracted research organizations performed these analyses.

### 3.7. Statistical Methods

A comparison of 4β-HC and 4β-HC/TC ratios before and after the administration of JTE-451 or JTE-761 was analyzed using the paired samples *t*-test. The AUC of midazolam was determined using non-compartmental analysis with Phoenix WinNonlin (version 6.2 or higher, Certara, Inc., Princeton, NJ, USA). The correlation between plasma 4β-HC levels (or 4β-HC/TC ratios) and the AUC of midazolam was assessed using Pearson’s correlation coefficient.

## Figures and Tables

**Table 2:** Summary of 4β-HC and TC concentrations, 4β-HC/TC ratios, the AUC of midazolam, and the CYP3A4 induction category obtained during the clinical DDI studies.

Parameter		JTE-451 200 mg BID (*n* = 16 ^#^)	JTE-451 400 mg BID (*n* = 18)	JTE-761 400 mg QD (*n* = 15 ^##^)
4β-HC concentration (ng/mL)	Before administration	22.7 ± 6.7	21.8 ± 9.5	34.1 ± 9.8
	After administration	25.0 ± 7.3	30.8 ± 13.0	45.6 ± 16.0
	% change (before vs. after)	11.8 ± 22.4	46.1 ± 28.9	33.6 ± 25.7
	Significance (before vs. after)	*p* = 0.059	*p* < 0.01	*p* < 0.01
TC concentration (mg/dL)	Before administration	177.9 ± 35.0	163.5 ± 27.0	181.9 ± 38.3
	After administration	157.6 ± 35.1	141.7 ± 26.5	145.0 ± 27.3
	% change (before vs. after)	−13.3 ± 13.1	−13.4 ± 6.9	−19.7 ± 7.7
4β-HC/TC ratio (×10^5^)	Before administration	1.28 ± 0.42	1.32 ± 0.50	1.93 ± 0.59
	After administration	1.65 ± 0.53	2.16 ± 0.73	3.13 ± 0.84
	% change (before vs. after)	29.3 ± 19.4	68.5 ± 30.6	68.1 ± 37.0
	Significance (before vs. after)	*p* < 0.01	*p* < 0.01	*p* < 0.01
Midazolam AUC (hr × ng/mL)	Before administration	39.2 ± 11.5	39.7 ± 14.9	40.8 ± 16.8
	After administration	36.3 ± 10.5	22.8 ± 7.9	18.9 ± 7.8
	% change (before vs. after)	−7.0 ± 18.8	−40.3 ± 11.7	−49.8 ± 20.9
CYP3A4 induction category		Very weak	Weak	Weak

The AUC of midazolam from time zero to the last quantifiable concentration was calculated from plasma concentrations at 0, 0.5, 0.75, 1, 1.5, 2, 3, 4, 6, 8, 12, 16, and 24 h after administration. Refer to Figure 3 for the calculation of other parameters. All values denote mean ± SD (*p* < 0.01). ^#^ Two subjects out of eighteen subjects opted out of the clinical study. ^##^ One subject out of sixteen subjects opted out of the clinical study.

## Data Availability

Not applicable.

## Supplementary Materials

The following supporting information can be downloaded at: https://www.mdpi.com/article/10.3390/molecules28041576/s1, Supplementary Figure S1: The dosing schedules of JTE-451 and JTE-761 in clinical DDI studies; Table S1: The parameters of LC-MS/MS and LC-HRMS.

## Sample Availability

Samples of the compounds are not available from the authors.
