# Correction: Taya et al. Clinical Evaluation Based on a New Approach to Improve the Accuracy of 4β-Hydroxycholesterol Measurement as a Biomarker of CYP3A4 Activity. *Molecules* 2023, *28*, 1576

**DOI:** 10.3390/molecules31183332

**Published:** 2026-09-20

**Authors:** Yuki Taya, Mari Mizunaga, Shunsuke Nakao, Mirinthorn Jutanom, Naoki Shimizu, Yukihiro Nomura, Kiyotaka Nakagawa

**Affiliations:** 1 Laboratory of Food Function Analysis, Graduate School of Agricultural Science, Tohoku University, Sendai 980-8572, Miyagi, Japan; yuki.taya@jt.com (Y.T.); jutanom.mirinthorn.d1@tohoku.ac.jp (M.J.); naoki.shimizu.c2@tohoku.ac.jp (N.S.); 2 Drug Metabolism and Pharmacokinetics Research Laboratories, Central Pharmaceutical Research Institute, Japan Tobacco Inc., Takatsuki 569-1125, Osaka, Japan; mari.mizunaga@jt.com (M.M.); shunsuke.nakao@jt.com (S.N.); yukihiro.nomura@jt.com (Y.N.)

In the original publication [1], the Conflicts of Interest statement was inaccurate. The correct Conflicts of Interest is as follows:

**Conflicts of Interest:** Yuki Taya, Mari Mizunaga, Shunsuke Nakao, and Yukihiro Nomura are employees of Japan Tobacco, Inc. Yuki Taya is also affiliated with Tohoku University for degree purposes. The authors declare that this study received funding from Akros Pharma, Inc., Princeton, NJ, USA. Akros Pharma, Inc. is a subsidiary of Japan Tobacco, Inc. Japan Tobacco, Inc. provided financial support for the conduct of the study. However, Japan Tobacco, Inc. was not involved in the study design, data collection, analysis, interpretation, writing of the manuscript, or the decision to submit it for publication.

The authors state that the scientific conclusions are unaffected. This correction was approved by the Academic Editor. The original publication has also been updated.
