# Supplementary material for: Clinical Evaluation Based on a New Approach to Improve the Accuracy of 4β-Hydroxycholesterol Measurement as a Biomarker of CYP3A4 Activity
Source: Molecules. 2023 Feb 7;28(4):1576. doi: 10.3390/molecules28041576 (PMC9967035; doi:10.3390/molecules28041576)
Supplement: Supplementary file 1 [file molecules-28-01576-s001.zip › molecules-2131623-supplementary.pdf]

molecules

Supplementary Materials

Clinical Evaluation Based on a New Approach to Improve the Accuracy of 4β-Hydroxycholesterol Measurement as a Biomarker of CYP3A4 Activity

Yuki Taya [1,2], Mari Mizunaga [2], Shunsuke Nakao [2], Mirinthorn Jutanom [1], Naoki Shimizu [1], Yukihiro Nomura [2] and Kiyotaka Nakagawa [1,*]

1 Laboratory of Food Function Analysis, Graduate School of Agricultural Science, Tohoku University, Sendai 980-8572, Miyagi, Japan; yuki.taya@jt.com (Y.T.); jutanom.mirinthorn.d1@tohoku.ac.jp (M.J.); naoki.shimizu.c2@tohoku.ac.jp (N.S.)

2 Drug Metabolism and Pharmacokinetics Research Laboratories, Central Pharmaceutical Research Institute, Japan Tobacco Inc., Takatsuki 569-1125, Osaka, Japan; mari.mizunaga@jt.com (M.M.); shunsuke.nakao@jt.com (S.N.); yukihiro.nomura@jt.com (Y.N.)

* Correspondence: kiyotaka.nakagawa.c1@tohoku.ac.jp; Fax: +81-22-757-4417

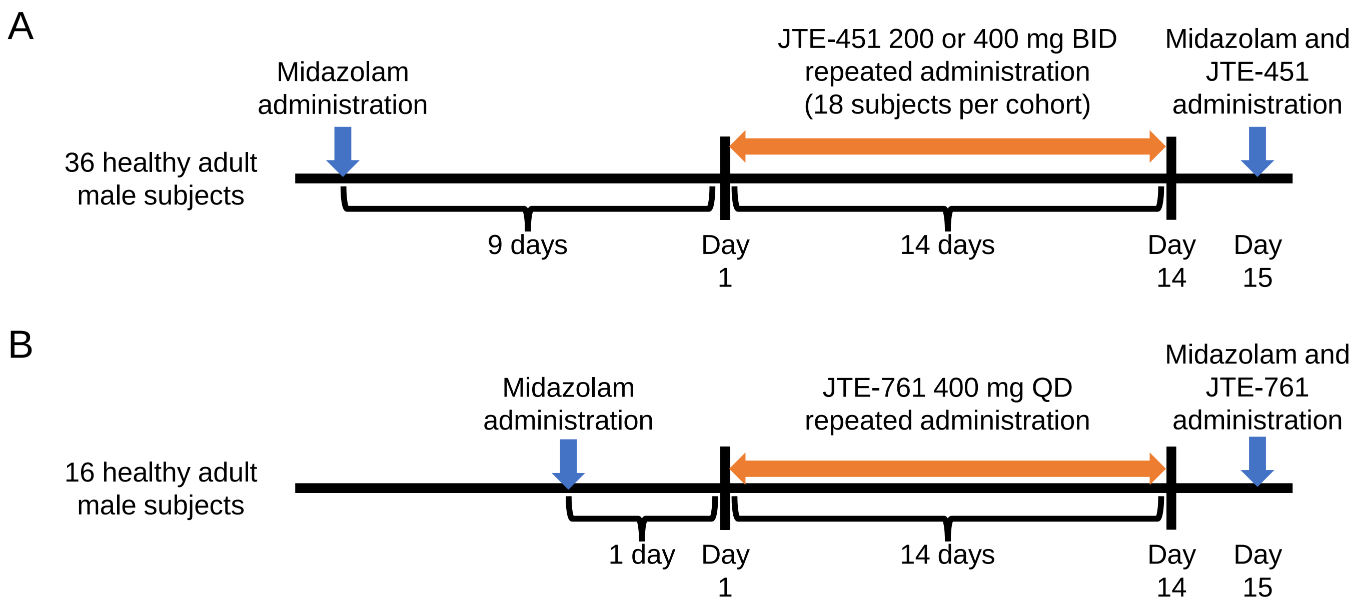

Figure S1. The dosing schedules of JTE-451 (A) and JTE-761 (B) in clinical DDI studies. The clinical DDI study of JTE-451 was conducted in 36 healthy adult male subjects. Thirty-six subjects were enrolled into two cohorts where subjects received either 200 or 400 mg BID dosing of JTE-451 (18 subjects per cohort). All subjects received a single oral dose of 3 mg midazolam 9 days prior to administration of JTE-451. Then, JTE-451 was administered for 14 consecutive days (Days 1 to 14). On Day 15, JTE-451 was co-administered with 3 mg midazolam. The clinical DDI study of JTE-761 was conducted in 16 healthy adult male subjects. Sixteen subjects were enrolled in a cohort where subjects received 400 mg QD dosing of JTE-761. All subjects received a single oral dose of 3 mg midazolam 1 day prior to administration of JTE-761. Then, JTE-761 was administered for 14 consecutive days (Days 1 to 14). On Day 15, JTE-761 was co-administered with midazolam.

Table S1. The parameters of LC-MS/MS and LC-HRMS.

Parameters	LC-MS/MS	LC-HRMS
Ion spray voltage (V)	4500	5500
Temperature (°C)	450	500
Curtain gas (psi)	30	25
Ion source gas 1 (psi)	60	50
Ion source gas 2 (psi)	80	50
Collision gas (psi)	8	-
